# Publisher Correction: Single-cell analysis uncovers fibroblast heterogeneity and criteria for fibroblast and mural cell identification and discrimination

**DOI:** 10.1038/s41467-020-18511-8

**Published:** 2020-09-03

**Authors:** Lars Muhl, Guillem Genové, Stefanos Leptidis, Jianping Liu, Liqun He, Giuseppe Mocci, Ying Sun, Sonja Gustafsson, Byambajav Buyandelger, Indira V. Chivukula, Åsa Segerstolpe, Elisabeth Raschperger, Emil M. Hansson, Johan L. M. Björkegren, Xiao-Rong Peng, Michael Vanlandewijck, Urban Lendahl, Christer Betsholtz

**Affiliations:** 1 Karolinska Institutet/AstraZeneca Integrated Cardio Metabolic Centre, Blickagången 6, SE-14157 Huddinge, Sweden; 2 Department of Medicine Huddinge, Karolinska Institutet, SE-14157 Huddinge, Sweden; 3 Department of Neurosurgery, Tianjin Medical University General Hospital, Tianjin Neurological Institute, Key Laboratory of Post-Neuroinjury, Neuro-Repair and Regeneration in Central Nervous System, Ministry of Education and Tianjin City, Tianjin, 300052 China; 4 Department of Immunology, Genetics and Pathology, Rudbeck Laboratory, Uppsala University, Dag Hammerskjölds väg 20, SE-75185 Uppsala, Sweden; 5 Klarman Cell Observatory, Broad Institute of MIT and Harvard, Cambridge, MA USA; 6 Icahn Institute for Genomics and Multiscale Biology, Department of Genetics and Genomic Sciences, Icahn School of Medicine at Mount Sinai, One Gustave L. Levy Place, New York, NY USA; 7 Bioscience Metabolism, Research and Early Development, Cardiovascular, Renal and Metabolism (CVRM) BioPharmaceuticals R&D, AstraZeneca, Gothenburg, Sweden; 8 Department of Cell and Molecular Biology, Karolinska Institutet, SE-17177 Stockholm, Sweden

**Keywords:** Transcriptomics, Cardiovascular biology

Correction to: *Nature Communications* 10.1038/s41467-020-17740-1, published online 07 August 2020.

The original version of this Article contained an error in the author affiliations.

Christer Betsholtz was incorrectly associated with ‘Department of Neurosurgery, Tianjin Medical University General Hospital, Tianjin Neurological Institute, Key Laboratory of Post-Neuroinjury, Neuro-Repair and Regeneration in Central Nervous System, Ministry of Education and Tianjin City, Tianjin 300052, China’ instead of the correct ‘Department of Immunology, Genetics and Pathology, Rudbeck Laboratory, Uppsala University, Dag Hammerskjölds väg 20, SE-75185 Uppsala, Sweden’.

This has now been corrected in both the PDF and HTML versions of the Article.

