# Supplementary material for: Single-cell analysis uncovers fibroblast heterogeneity and criteria for fibroblast and mural cell identification and discrimination
Source: Nat Commun. 2020 Aug 7;11:3953. doi: 10.1038/s41467-020-17740-1 (PMC7414220; doi:10.1038/s41467-020-17740-1)
Supplement: Supplementary file 1 — Supplementary Information [file 41467_2020_17740_MOESM1_ESM.pdf]

Supplementary Information
for

Single-cell analysis uncovers fibroblast heterogeneity and criteria for fibroblast and mural cell identification and discrimination

(Muhl et al.)

Supplementary Figure 1:

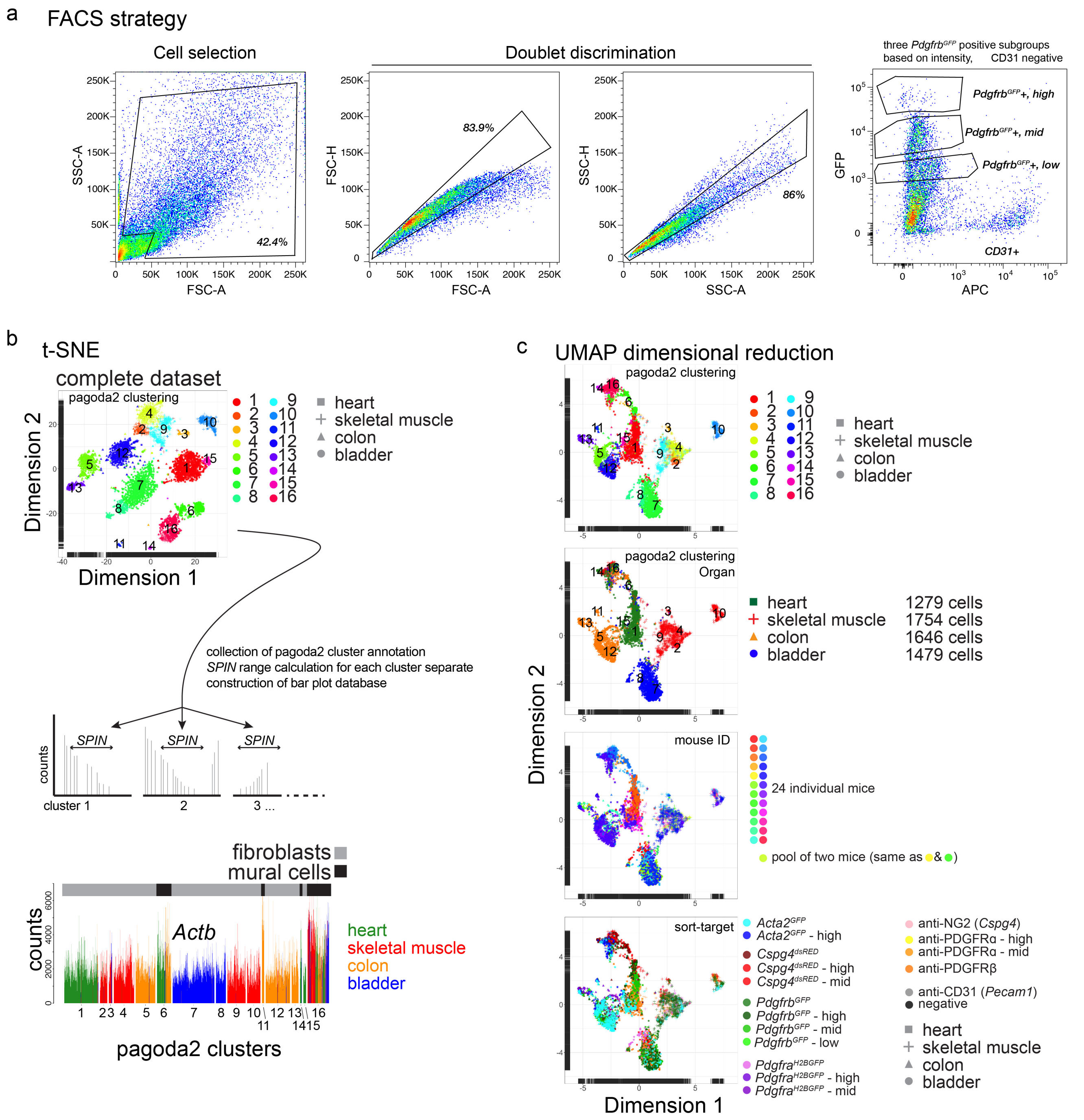

Supplementary Figure 1: **Cell sorting and clustering.**

a, Representative FACS plots indicating cell selection. Cells were first broadly selected for singlet qualities (doublet discrimination) based on area and complexity, and then selected based on fluorescent reporter gene expression or antibody-based fluorescent signal. The example here shows cells from *Pdgfrb*^{GFP} colon, co-stained with anti-CD31 antibody to identify potential doublets between mesenchymal cells (*Pdgfrb*$^{GFP+}$) and endothelial cells (CD31; *Pecam1*$^{+}$). Right panel shows gates selected for different *Pdgfrb*^{GFP} reporter intensity. **b**, Strategy for bar-plot generation: Pagoda2 clusters (shown with *t*-distributed stochastic neighbor embedding (*t*-SNE) visualization) were used to group the cells for the bar plot database. The cell ordering within each cluster was done by SPIN, which places the most closely similar cells next to each other. The resulting bar plot, here represented by *Actb* (α-actin) mRNA, is colour-coded for organ of origin (heart, green; skeletal muscle, red; colon, orange; bladder, blue). Fibroblasts clustered into twelve clusters (# 1-5, 7-10, 12, 13 and 15), while mural cells clustered together in four clusters (# 6, 11, 14 and 16). **c**, UMAP visualizations of the complete dataset with pagoda2 clusters colour coded and annotated (first panel), organ of origin of each cell colour coded and pagoda2 clusters annotated (second panel), individual mice as origin of each cell colour coded (third panel) (see also Supplementary Table 4), and the targeting strategy used for FACS of each cell colour coded (fourth panel).

Supplementary Figure 2:

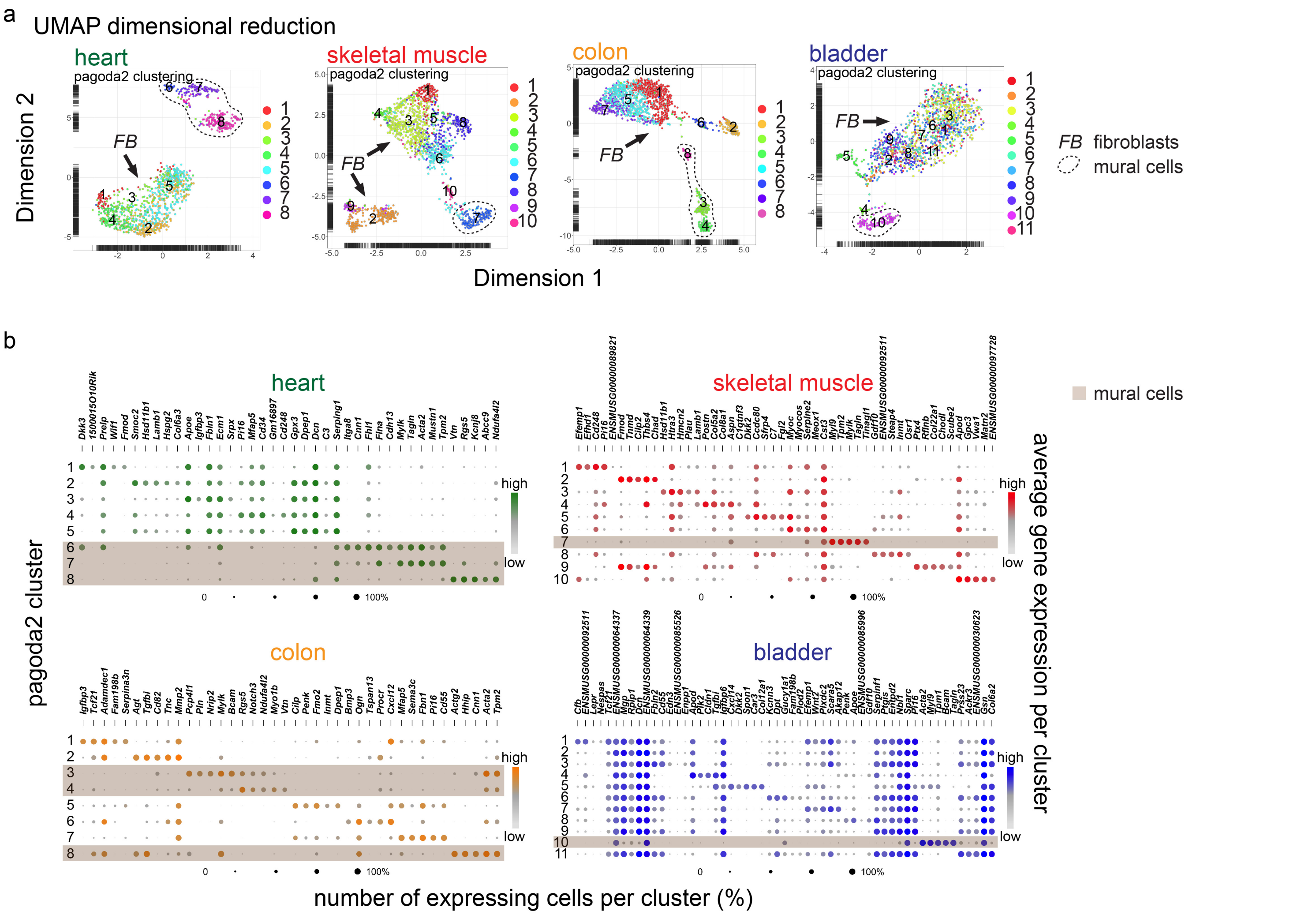

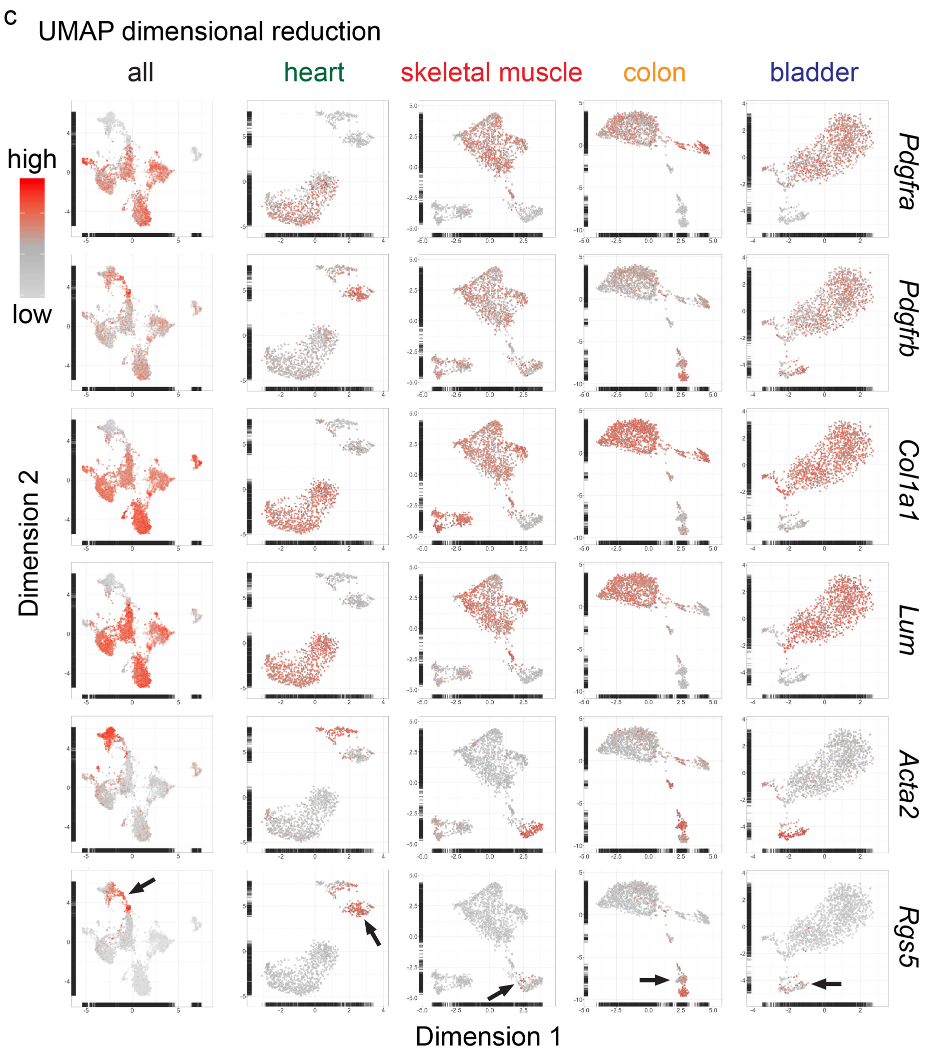

<u>Supplementary Figure 2:</u>

Cell type classification.

a, UMAP visualization of the pagoda2 clustering for each organ dataset separately (compare to Fig. 1d). The pagoda2 clusters are colour coded and mural cell and fibroblast clusters indicated. **b**, Dot plot of the top 5 enriched genes per cluster for each of the organ datasets (grey: low, respective colour: high expression; heart, green; skeletal muscle, red; colon, orange; bladder, blue). Dot size indicated percentage of cells per cluster expressing the respective gene. Clusters containing mural cells are indicated by a grey horizontal shadow. **c**, UMAP visualization of marker gene expression (grey, low; red, high) in the complete dataset and single organ datasets. In all single organ datasets, a broad dispersion of fibroblasts was visible, with the clearest distinction into two separate clouds seen in the colon dataset. Cell clouds containing mural cells are indicated in the plots showing *Rgs5* expression. Note the absence of *Pdgfra* expression in mural cell clouds.

Supplementary Figure 3:

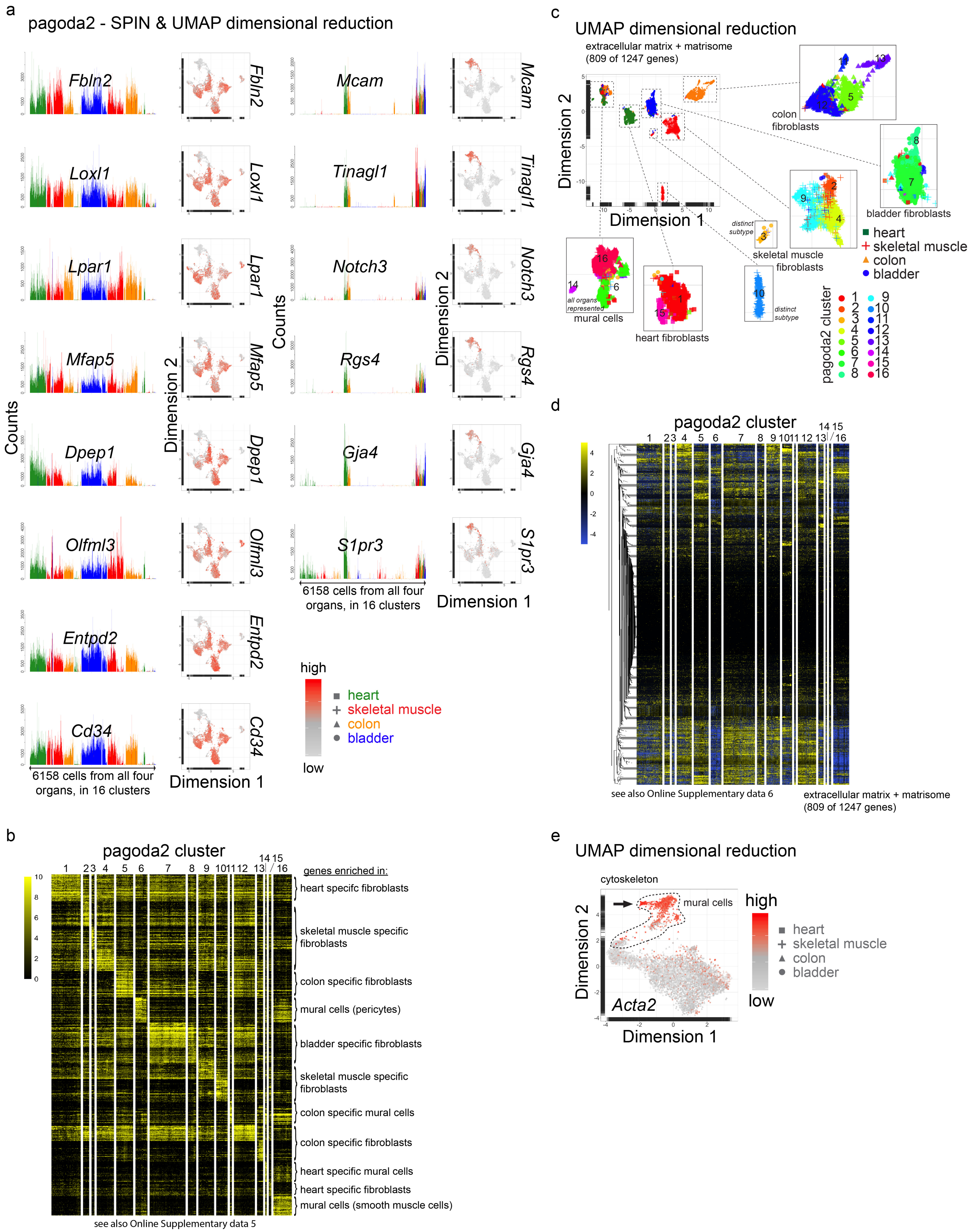

Supplementary Figure 3: **Organotypicity is determined by expression of ECM components.**

a, Bar plot and UMAP visualization of global fibroblast-enriched (*Fbln2*, *Loxl1*, *Lpar1*, *Mfap5*, *Dpep1*, *Olfml3*, *Entpd2*, *Cd34*) or mural cell-enriched (*Mcam*, *Tinagl1*, *Notch3*, *Rgs4*, *Gja4*, *S1pr3*) genes in the complete dataset (grey, low; red, high). Note the higher dispersion clustering of fibroblasts in comparison with the mural cell clusters. **b**, Expression heat map (black, low; yellow, high) showing pagoda2 cluster over-represented genes that were selected by differential gene expression analysis of the respective cluster against the remaining dataset (see also Online Supplementary data 5). **c**, UMAP visualization of ECM+matrisome genes (809 genes were expressed in the complete dataset, out of 1247 genes compiled in the ECM+matrisome gene list, see also Fig. 2c), color-coded for organ of origin (main UMAP plot), or superimposed pagoda2 clusters in the magnification frames. Annotation of cell clouds from the ECM+matrisome UMAP analysis was done considering cellular organ-origin and cluster allocations. UMAP analysis with the restricted ECM+matrisome gene set resulted in the most distinct separation, especially of the fibroblasts. Of note; fibroblasts retained organ-specific clustering. This observation suggests that ECM components are major distinguishing molecular factors of fibroblast subtypes between organs, but also within organs as specific subclasses of fibroblasts were separated, as shown in skeletal muscle (annotation: distinct subtype). **d**, Expression heat map of all 809 genes in the ECM+matrisome gene-set (blue, low; yellow, high) (see also Online Supplementary data 6). **e**, UMAP visualization of ‘cytoskeleton’ GO-associated genes (1785 genes were expressed in the complete dataset, out of 2068 genes compiled in the ‘cytoskeleton’ GO-term, see also Fig. 2c), colour coded for the expression of *Acta2* (grey, low; red, high); it became evident that cells found in the separated cloud in this UMAP analysis were enriched in *Acta2* expression (arrow), suggesting that genes of the cytoskeleton GO-term are major determining factors for identity of SMC and possible other *Acta2*$^{+}$ cells.

Supplementary Figure 4:

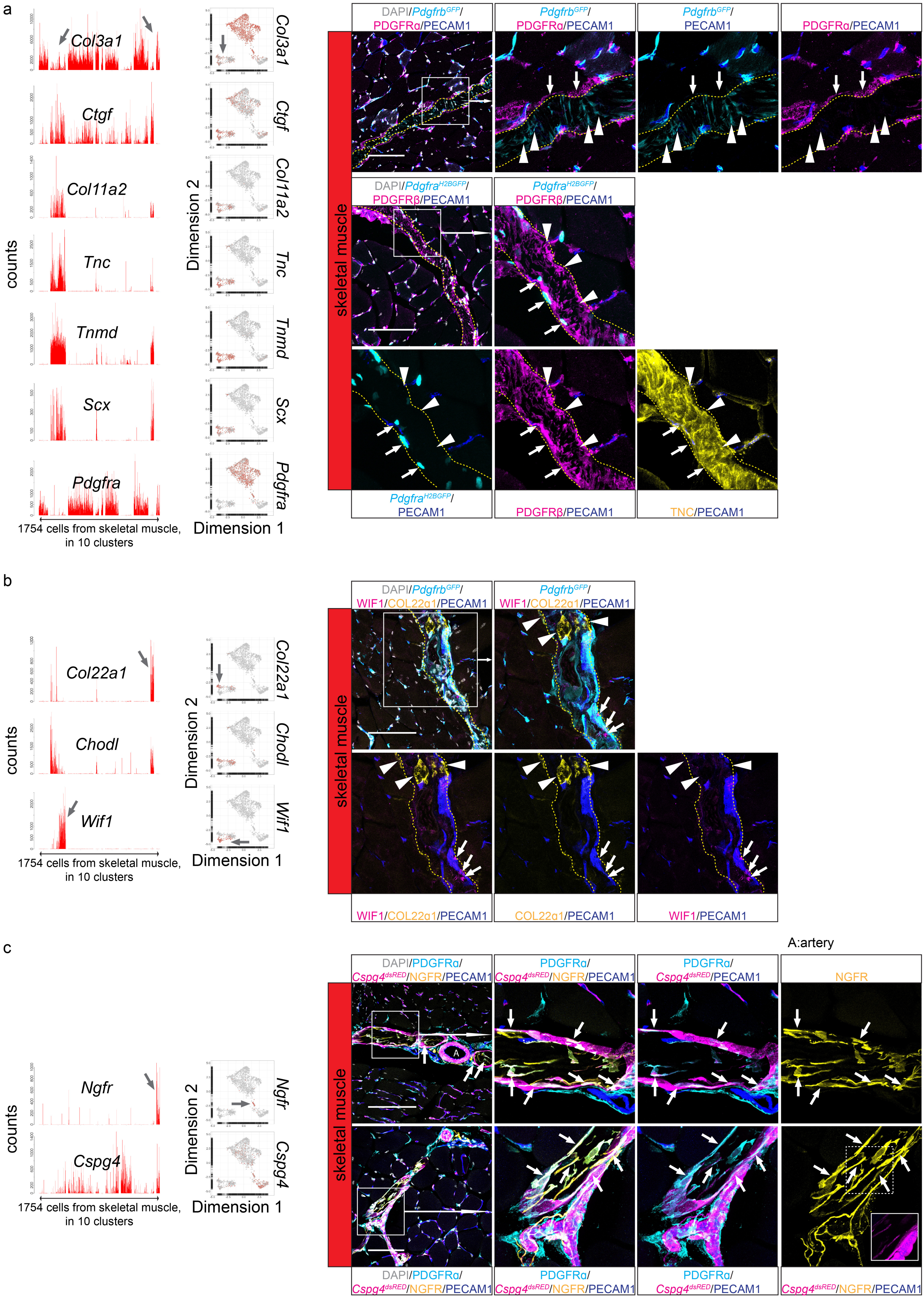

Supplementary Figure 4: **Mapping of fibroblast subtypes in the skeletal muscle.**

a, Left: Bar plots and UMAP visualization showing examples of genes specifically expressed in the perimysial cell clusters (*Ctgf*, *Col11a2*, *Tnc*, *Tnmd*, *Scx*) in the skeletal muscle dataset, and *Col3a1*, which is expressed to a lower extent in the perimysial cells. Note the low expression of *Pdgfra* in the perimysial cell clusters. Right: Immunofluorescence staining of skeletal muscle from $Pdgfrb^{GFP}$ (upper panel) or $Pdgfra^{H2BGFP}$ (lower panel) reporter lines for the indicated markers showed that perimysial cells exhibit low expression and protein levels of PDGFRα, however perimysial cells are positive for PDGFRβ protein and gene expression (arrowheads). $Pdgfra^{+}$ cells instead were found directly adjacent to the perimysial fascia (arrows) and in the endomysium. Scale bars: 100 µm. **b**, Left: Bar plots and UMAP showing examples of differentially expressed genes within the perimysial cell clusters (*Col22a1*, *Chodl*, *Wif1*) of the skeletal muscle dataset. Right: Immunofluorescence staining confirmed the differential presence of type-XXII collagen α-1 (arrowheads) and WIF1 (arrows) within the perimysial fascia. Scale bar: 100 µm. **c**, Left: Bar plots and UMAP showing the pagoda2 cluster 10 (nerve fibre fibroblast) enriched gene; *Ngfr* (arrow). Note the co-expression of *Cspg4* and *Pdgfra* in cluster 10. Right: Immunofluorescence staining of skeletal muscle from $Cspg4^{dsRED}$ reporter line for the indicated markers shows the presence of triple positive (NGFR, $Cspg4^{dsRED}$, PDGFRα) cells at the border of nerve fibres (arrows). A: artery. Scale bars: 200 µm (upper panel), 100 µm (lower panel). UMAP visualization (**a**-**c**) gene expression scale: grey, low; red, high.

Supplementary Figure 5:

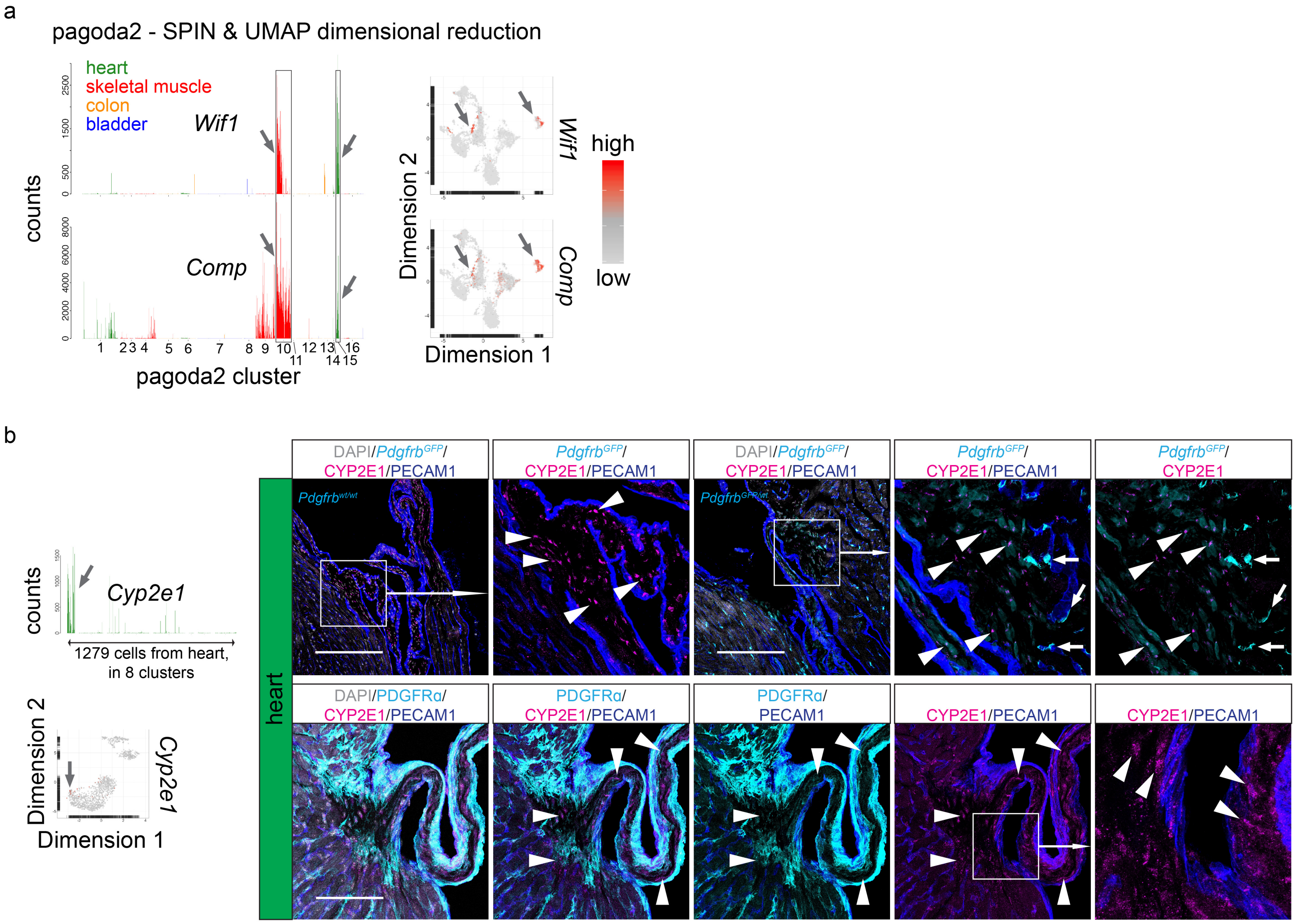

Supplementary Figure 5: **Mapping of fibroblast subtypes in the heart.**

a, Bar plots and UMAP visualization of the complete dataset showing co-expression of perimysial genes (*Wif1* and *Comp*) in a subset of cells captured from the heart (arrows) of cell subtype specifically expressed genes. **b**, Left: Bar plot and UMAP (grey, low; red, high expression) of *Cyp2e1* that is specifically enriched in cardiac valve interstitial cells. Right: Immunofluorescence staining of the cytochrome P450 member (CYP2E1) in the heart of *Pdgfrb*^{GFP} reporter line, confirming the presence of the protein in cardiac valve structures (arrowheads). *Pdgfrb*^{GFP}-negative littermate control sample (*Pdgfrb*$^{wt/wt}$) shows the specificity of the reporter construct (*Pdgfrb*$^{GFP/wt}$). PDGFRα staining revealed a fibroblast population of high PDGFR α signal that seems to encompass the stroma of the hinge region that extends also along the valve leaflets. Scale bars: 200 µm (upper panel), 100 µm (lower panel).

Supplementary Figure 6:

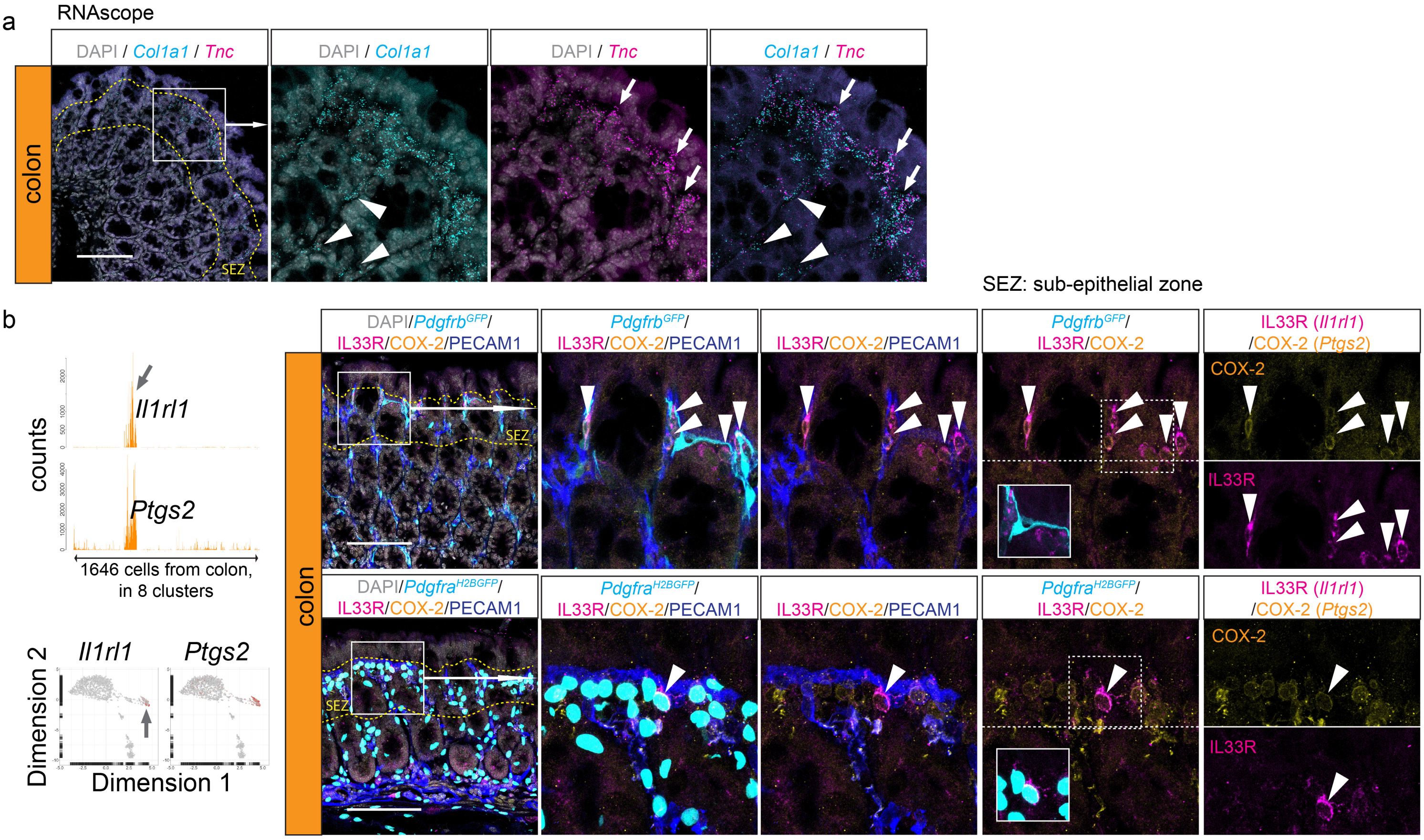

<u>Supplementary Figure 6:</u> **Mapping of crypt apex fibroblast subtypes in the colon.**

a, RNAscope fluorescence staining for *Col1a1* (cyan) and *Tnc* (magenta), pinpointing the location of Tnc^{+} $Cd34^{-}$ cells at the top of the colonic crypt (arrows), whereas *Col1a1* expressing fibroblasts are also found at deeper levels in the colon mucosa (arrowheads). Scale bar: 100 µm. **b**, Left: Bar plots and UMAP visualization (grey, low; red, high expression) showing specific expression of *Il1rl1* and *Ptgs2* in the Tnc^{+} $Cd34^{-}$ fibroblast subtype from the colon. Right: Immunofluorescence staining for the IL33R (*Il1rl1*) and COX-2 (*Ptgs2*) proteins in colon samples from $Pdgfrb^{GFP}$ (upper panel) or $Pdgfra^{H2BGFP}$ (lower panel) reporter lines confirmed the location of these cells at the colonic surface. Arrowheads: (upper panel) IL33R / COX-2 double positive cells are not pericytes (high $Pdgfrb^{GFP}$ signal); (lower panel) IL33R / COX-2 double positive cells are also positive for $Pdgfra^{H2BGFP}$. Scale bars: 100 µm.

Supplementary Figure 7:

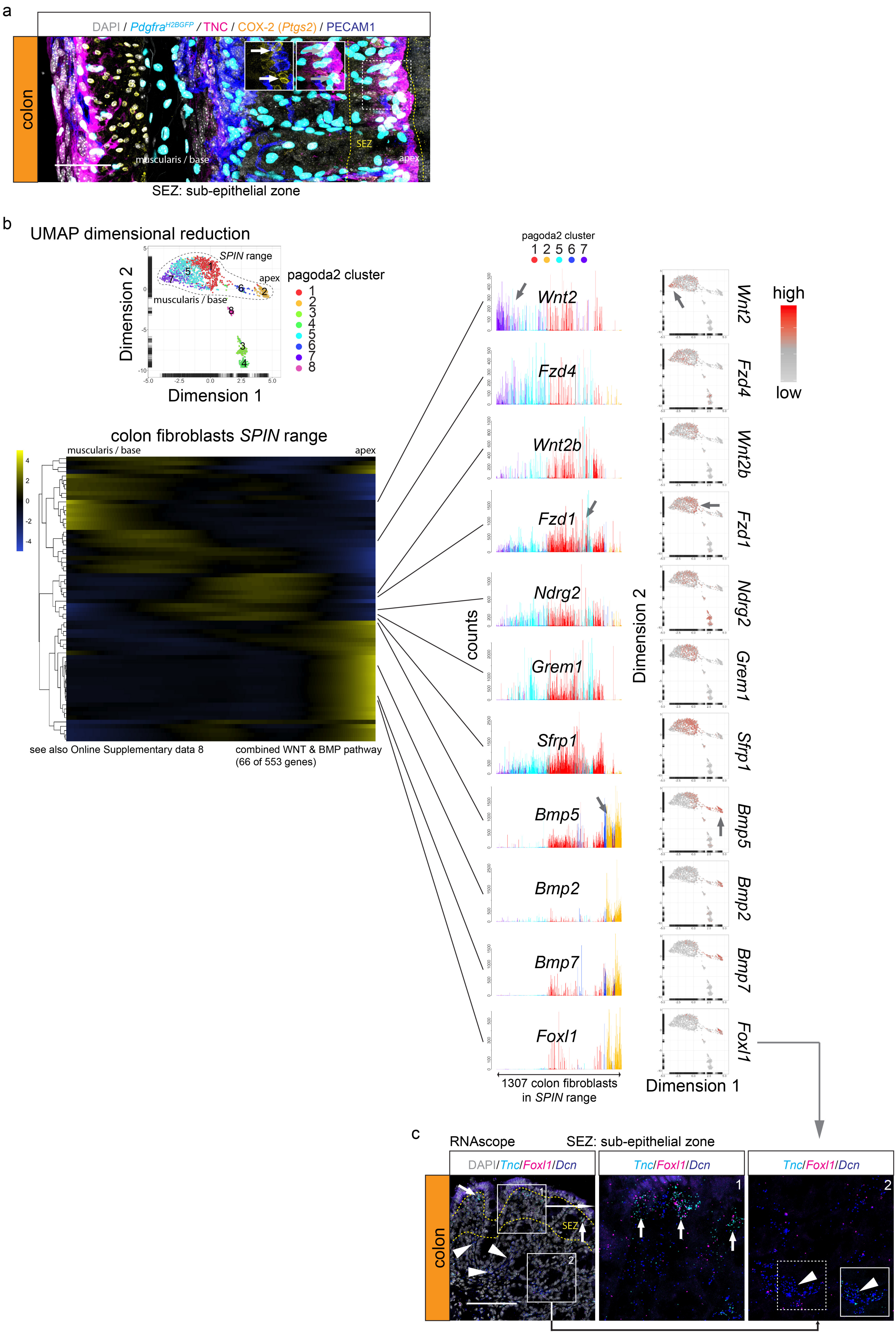

Supplementary Figure 7: **Zonation of fibroblast subtypes in the colon.**

a, Immunofluorescence staining of colon from *Pdgfra*^{H2BGFP} reporter line for TNC, COX-2 and PECAM1, showing *Pdgfra*^{H2BGFP}/COX-2 double positive cells in the TNC^{+} sub-epithelial zone, confirming the presence of the *Pdgfra*$^{+}$ *Tnc*$^{+}$ *Ptgs2*$^{+}$ *Cd34*$^{-}$ cell population close to the colonic apex surface. Scale bar: 50 μm. **b**, Upper: UMAP visualization highlighting colon fibroblast populations analysed by SPIN (pagoda2 clustering colour coded and annotated). Lower: Expression heat map (loess smoothed values; blue, low; yellow high) of a gene-set compiled from genes assigned to 'WNT signaling pathway' (GO:0016055) or 'BMP signaling pathway' (GO:0030509) that were expressed with a sum of at least 1000 counts in the fibroblasts of the colon, and exhibited differential expression within the SPIN range (66 out of 553 genes). The order of cells in the heat map is according to the colon fibroblast SPIN analysis, and we suggest that this order reflects the zonation of fibroblasts along the crypt-surface axis of the colonic crypt (left, muscularis/base; right, apex) (see also Online Supplementary data 8). Bar plots from SPIN analysis (colour coding retained from pagoda2 clustering) of the colon show examples of genes expressed in a zonated fashion along the crypt-surface axis of the colonic crypt, arrows indicate the respective cell subclasses. UMAP visualization of colon dataset highlighting the zonated distribution of cells (arrows) also in the UMAP landscape. **c**, RNAscope of *Tnc* (cyan), *Foxl1* (magenta) and *Dcn* (blue) expression to pinpoint the location of *Tnc*$^{+}$ *Foxl1*$^{+}$ cells close to crypt apex surface, confirming the zonation along the crypt-surface axis. Scale bar: 100 μm. SEZ: sub-epithelial zone.

Supplemental Figure 8:

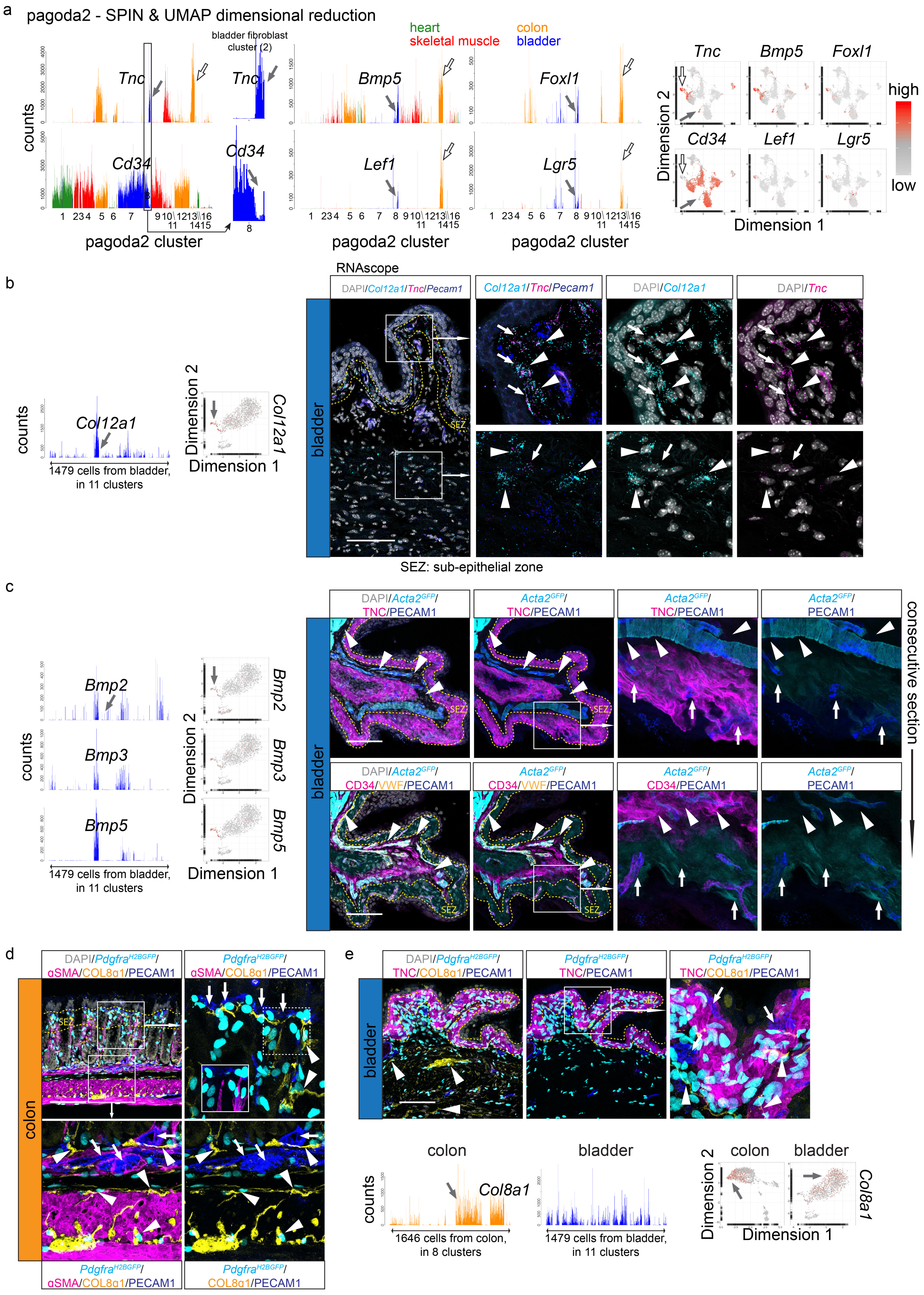

Supplementary Figure 8: **Mapping of fibroblast subtypes in the bladder.**

a, Bar plots and UMAP visualization of pagoda2 analysis of the complete dataset illustrating the presence of *Tnc*$^{+}$ *Cd34*$^{-}$ fibroblasts also in the bladder (in pagoda2 cluster 8) and examples of co-enriched genes in these specific fibroblasts of the colon (open arrows) and bladder (arrows), such as *Bmp5*, *Foxl1*, *Lef1* and *Lgr5*. **b**, Left: Bar plot and UMAP of *Col12a1* in bladder pagoda2 analysis. Right: RNAscope staining for *Col12a1* (cyan), *Tnc* (magenta) and *Pecam1* (blue), pinpointing the location of *Col12a1*$^{+}$ *Tnc*$^{+}$ (*Cd34*$^{-}$) cells to the sub-epithelial zone of the bladder (arrows), while some *Col12a1*$^{+}$ cells were also found in deeper regions of the bladder mucosa and detrusor muscularis (arrowheads), in accordance with the broader expression pattern of *Col12a1* in the bladder dataset, compared to *Tnc* (Fig. 5b) Scale bar: 100 µm. **c**, Left: Bar plots and UMAP of BMP ligand genes (*Bmp2*, *Bmp3*, *Bmp5*) in the bladder pagoda2 analysis, specifically enriched in the *Tnc*$^{+}$ *Cd34*$^{-}$ fibroblast subtype (arrows). Right: Immunofluorescence staining of bladder samples from *Acta2*^{GFP} reporter line, demonstrating *Acta2* expression in *Tnc*$^{+}$ *Cd34*$^{-}$ fibroblasts (arrows). Arrowheads: *Tnc*$^{-}$ *Cd34*$^{+}$ fibroblasts co-localizing with larger arteriolar vessels (perpendicular vessels). Upper and lower panel are consecutive sections. Scale bars: 100 µm. **d**, **e**, Immunofluorescence staining of colon sample (**d**) or bladder sample (**e**) from *Pdgfra*^{H2BGFP} reporter line, stained for αSMA (colon) or TNC (bladder) together with type-VIII collagen α-1 (COL8α1) and PECAM1. Arrowheads highlight COL8α1 positive structures within the colon muscularis as well as mucosa, or the bladder detrusor muscularis and mucosa. Arrows point to vascular structures. **e**, lower panel, Bar plot of colon or bladder pagoda2 analysis show expression patterns of *Col8a1* in the respective organ. Note the absence of *Col8a1* in *Acta2*$^{+}$ cell clusters (compare to Fig. 1c), despite close proximity of type-VIII collagen α-1 staining with αSMA positive cells in the colon mucosa, muscularis mucosae, or bladder detrusor. UMAP visualization (**b**, **c**, **e**) gene expression scale: grey, low; red, high. Scale bars: 100 µm.

Supplementary Figure 9:

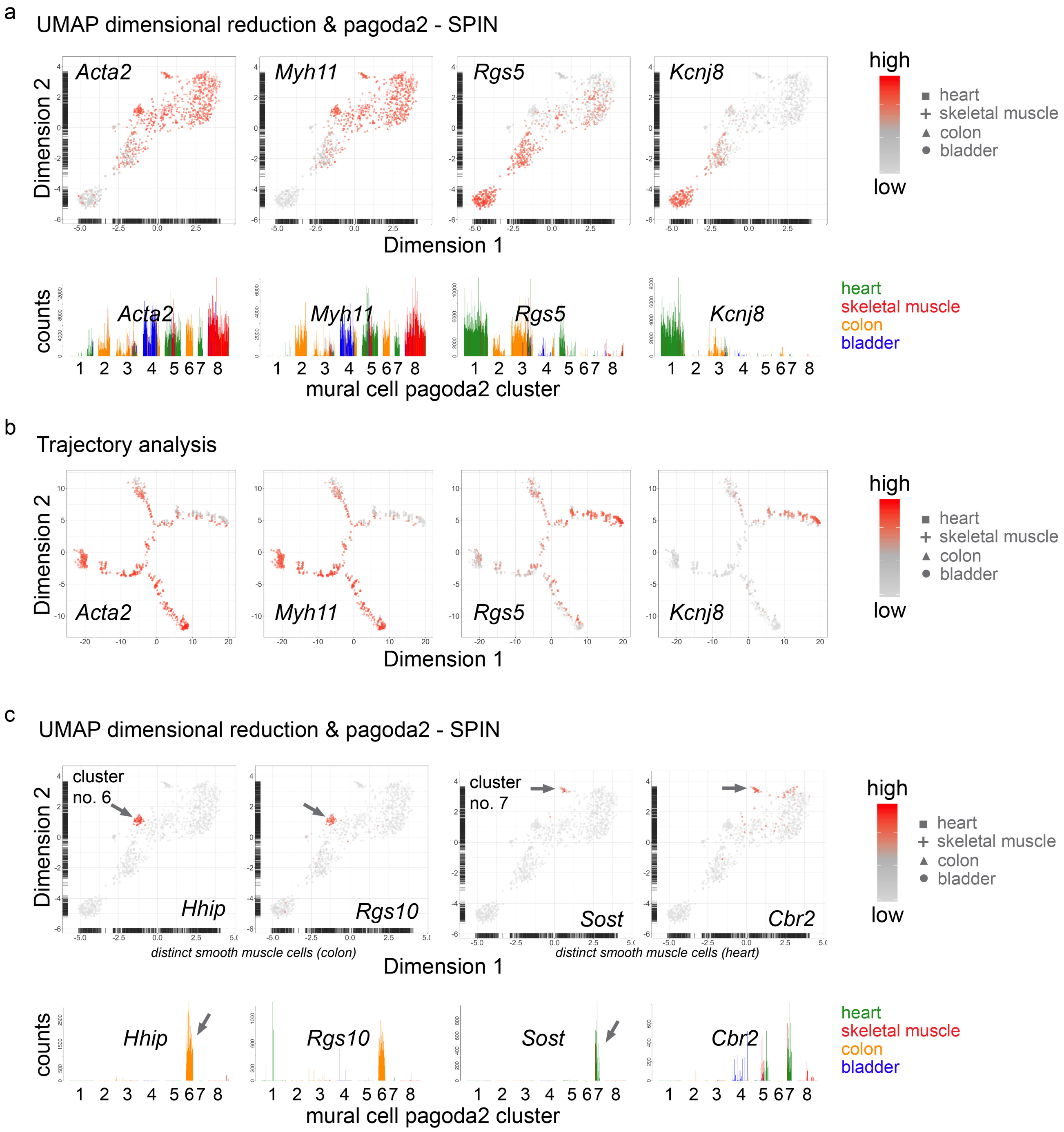

Supplementary Figure 9: **Mural cell heterogeneity.**

a, UMAP and bar plot visualization of the mural cell dataset showing expression intensity of *Acta2*, *Myh11*, *Rgs5* and *Kcnj8*. The identified clusters are indicated. **b**, Gene expression visualization of *Acta2*, *Myh11*, *Rgs5* and *Kcnj8* superimposed on the trajectory analysis landscape. Similar to the UMAP landscape, the expression of marker genes for pericytes or SMC, respectively, appeared in a gradual manner, also in the trajectory landscape. **c**, UMAP and bar plots of genes enriched in organ-specific mural cell clusters (arrows); *Hhip* and *Rgs10* in the colon-specific subtype (cluster 6), *Sost* and *Cbr2* in the heart-specific subtype (cluster 7).

Supplementary Figure 10:

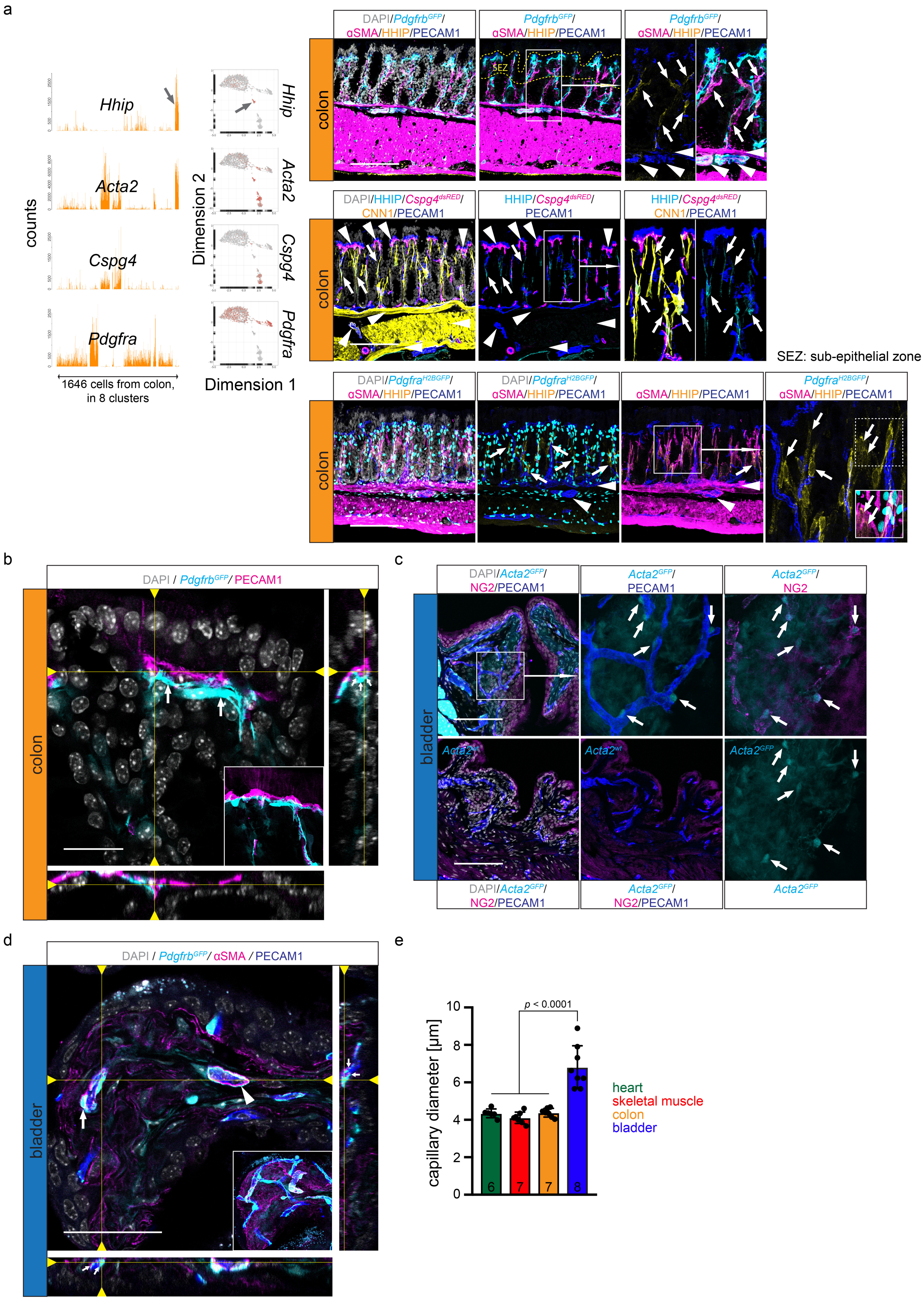

Supplementary Figure 10: **Organ-specific mural cell subtypes.**

a, Left: Bar plots and UMAP visualization (grey, low; red, high expression) of genes enriched in the interstitial SMC (*Hhip*, *Acta2*) of the colon. Note the absence of *Cspg4* and *Pdgfra* expression. Right: Immunofluorescence staining of colon samples from $Pdgfrb^{GFP}$ (upper), $Cspg4^{dsRED}$ (middle) or $Pdgfra^{H2BGFP}$ (lower) reporter lines for HHIP and PECAM1 together with αSMA (upper and lower panel) or CNN1 (middle panel). Arrows highlight $HHIP^{+}$ αSMA^{+} $CNN1^{+}$ interstitial SMC that are negative for *Cspg4* and *Pdgfra*. Arrowheads indicate αSMA^{+} $CNN1^{+}$, but $HHIP^{-}$ vascular SMC and $Cspg4^{+}$ pericytes (middle panel). Scale bars: 100 µm. SEZ: sub-epithelial zone. **b**, Orthogonal views of immunofluorescence staining of colon sample from $Pdgfrb^{GFP}$ reporter line for PECAM1. Arrows highlight pericytes located at the opposing, abluminal side of the sub-epithelial capillary loop. Inlay shows maximum intensity projection. Scale bar: 25 µm (see also Supplementary Movie 1, 2). **c**, Immunofluorescence staining in bladder samples from the $Acta2^{GFP}$ reporter line stained for NG2 (*Cspg4*) and PECAM1. Arrows indicate $Acta2^{+}$ pericytes located at the capillaries of the sub-epithelial plexus. $Acta2^{GFP}$-negative littermate control shows the specificity of the reporter construct. Scale bar: 100 µm. **d**, Orthogonal views of immunofluorescence staining of bladder sample from $Pdgfrb^{GFP}$ reporter line for αSMA and PECAM1. Arrows highlight $Acta2^{+}$ pericyte. Arrowhead: larger perpendicular vessel with SMC coat. Inlay shows maximum intensity projection. Scale bar: 50 µm. **e**, Quantification of capillary diameter in all four organs, showing the significantly higher diameter of bladder capillaries in comparison to capillaries from the heart, skeletal muscle and colon (excluding sub-epithelial capillary loop). Number of animals (n) is given in the bar graph, *p*-value was calculated using one-way ANOVA with Tukey's multiple comparison test, and is given in the bar graph (single adjusted *p*-values: heart vs. bladder $p = 0.0000019157$, skeletal muscle vs. bladder $p = 0.00000019628$, and colon vs. bladder $p = 0.000001189$). Error bars represent SD.

Supplementary Figure 11:

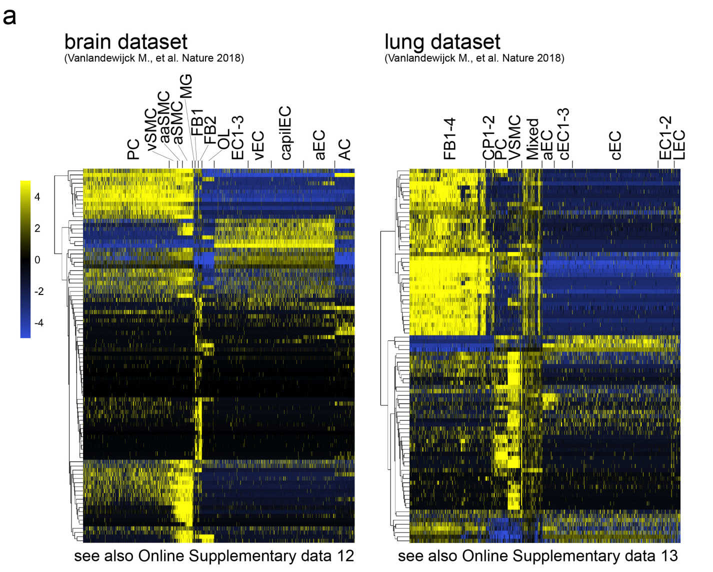

Supplementary Figure 11:

Fibroblast and mural cell gene signature application.

a, Expression heat maps (blue, low; yellow, high) of genes compiled in the fibroblast or mural cell enrichment signatures across heart, skeletal muscle, colon and bladder (see Fig. 1f) in the entire brain (left) or lung (right) datasets [14] (see also Online Supplementary data 12, 13). **b**, **c**, Expression heat maps (blue, low; yellow, high) of genes compiled in the fibroblast or mural cell enrichment signatures applied on the Tabula Muris data collection [53], showing 'limb muscle' droplet and FACS (**b**, left), 'heart & aorta' droplet and 'heart' FACS (**b**, right), 'bladder' droplet and FACS (**c**, left) and 'lung' droplet and FACS (**c**, right) datasets (see also Online Supplementary data 14-21). Note the cluster specific expression of signature genes, indicating the ability to identify fibroblast as well as mural cell populations in unknown datasets, as well as the partial expression of mural cell signature genes in endothelial cells.

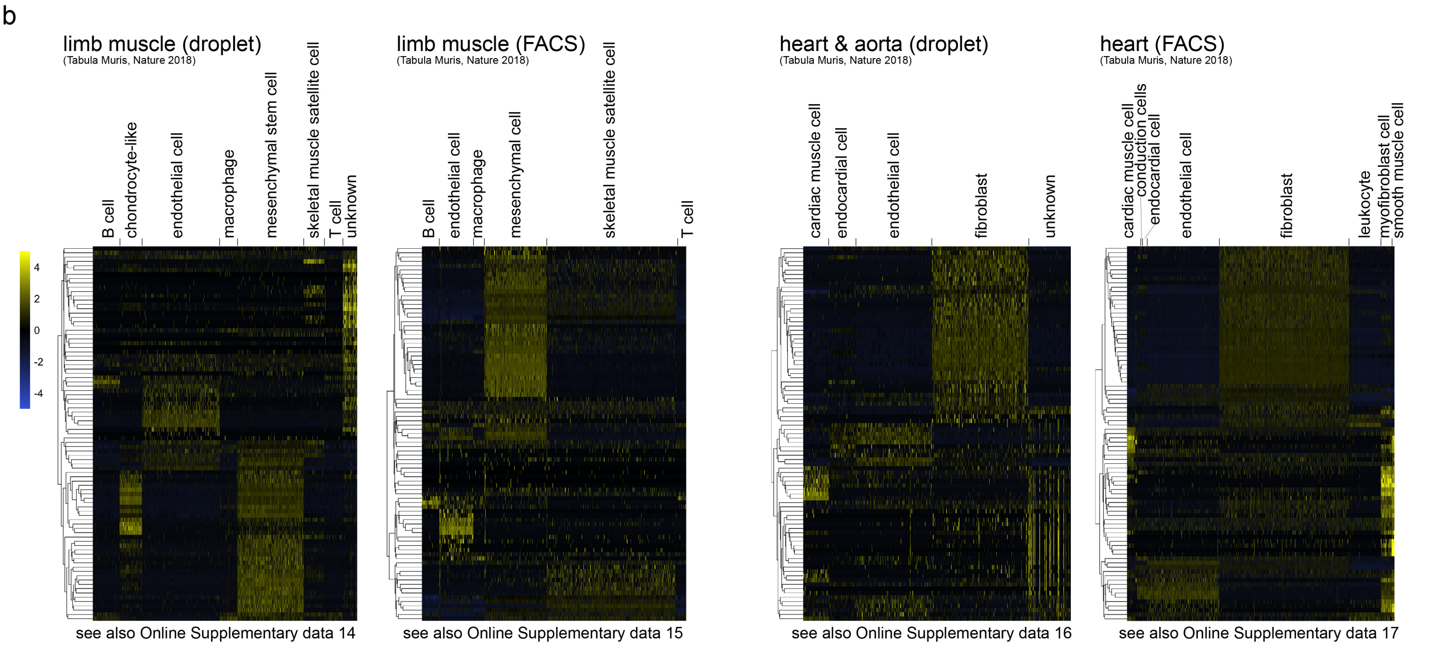

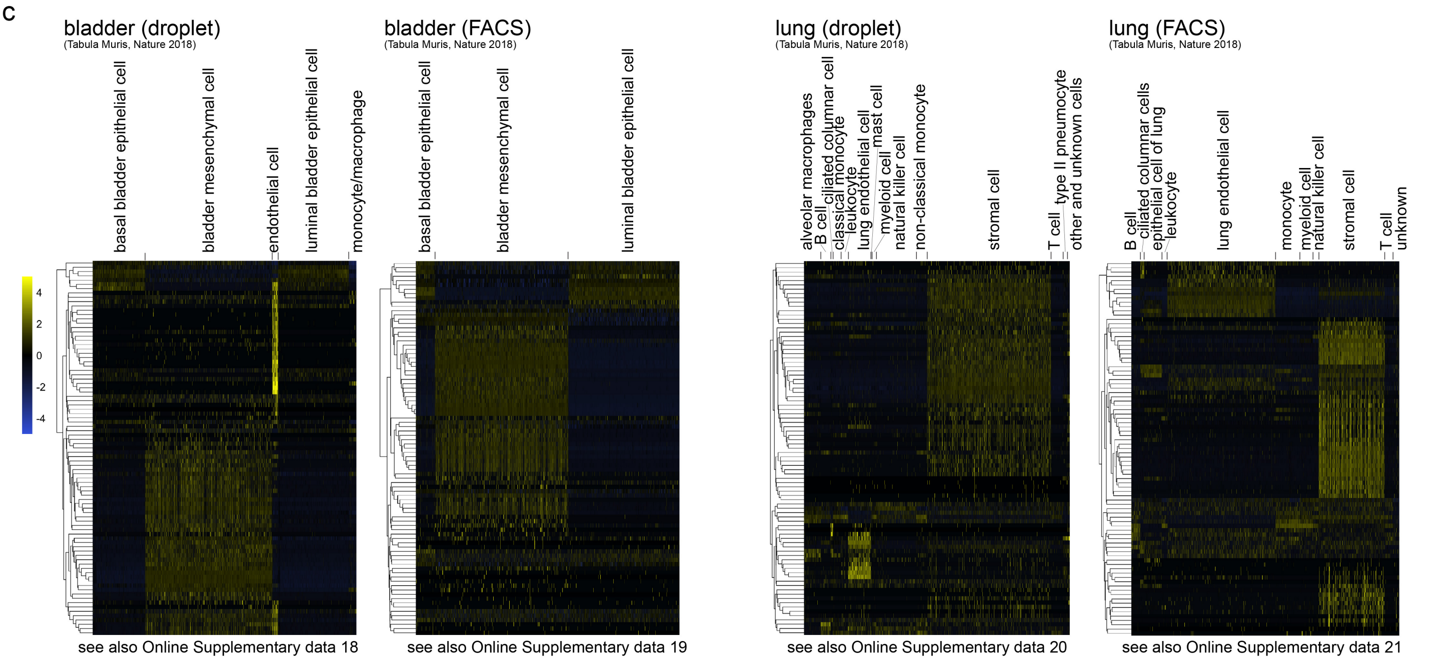

Supplementary Table 1: **Inter-organ cross-reference of fibroblasts vs mural cell enriched genes per organ.**

differential expression
log2 FC >2

organs	heart	skeletal muscle	colon	bladder	heart & skeletal muscle	heart & colon	heart & bladder	skeletal muscle & colon	skeletal muscle & bladder	colon & bladder	heart & skeletal muscle & colon	heart & skeletal muscle & bladder	heart & colon & bladder	skeletal muscle & colon & bladder	heart & skeletal muscle & colon & bladder
Fibroblast genes	*Lamc1, Glul, Fibin, Gpx7, Hspg2, Cxcl1, Olfml1, Lpl, Klf2, Crispld2, ENSMUSG00000090639, Trf, Meox1, Jpt1, Pdia6, ENSMUSG00000021871, Tmem45a, Aldh1a1, Gpm6b*	*Idh1, Fmod, Prg4, Angptl1, Myoc, Olfml2b, Fkbp7, Rin2, Sec24d, Il11ra1, Steap4, Fam114a1, Antxr2, Tmem119, Fkbp9, Capg, C1ra, Plekhf1, ENSMUSG00000030804, Vkorc1, Cpxm2, Cmtm3, Nrp1, Col5a3, Rora, Lysmd2, Amotl2, Epb41l2, Marcks, Gja1, P4ha1, Nfic, Rap1b, Egfr, Ccl11, Lasp1, Jup, Fkbp10, Abca8b, Abca9, Mxra7, Socs3, Gaa, Frmd6, Cxcl14, Thbs4, Il6st, Plau, Dpysl2, Dap, Kdelr3, Cebpd, Fstl1, Pros1, Tapbp, Trip10, Jcad, Nrep, Pcdhga1, Pcdhga2, Pcdhga3, Pcdhgb1, Pcdhga4, Pcdhgb2, Pcdhga5, Pcdhga6, Pcdhga7, Pcdhgb4, Pcdhga8, Pcdhgb5, Pcdhga9, Pcdhgb6, Pcdhga10, Pcdhgb7, Pcdhga11, Pcdhgb8, Pcdhga12, Pcdhgc4, Pcdhgc5, Ppic, Ahnak, Lamp2, Tsc22d3*	*Igsf10, Fam198b, Tnc, Lepr, Hpse, Eln, Igfbp3, Slit3, Adamdec1, C4b, Pcsk5*	*Dner, Rab7b, Plekha6, Cfh, Pla2g4a, Mpzl1, Creg1, Pbx1, Uap1l1, Npdc1, Ptges, Angptl2, Sqor, Prnp, Thbd, Pltp, Tshz2, Pcdh18, Rnf13, Kcnn3, Slc39a1, Sigmar1, Ece1, Nbl1, H6pd, Mn1, Por, Wnt2, Pdia4, Gpnmb, Cxcl12, Tm7sf3, Ppfibp1, Aldh1a3, Akap13, Serpinh1, Arap1, Dkk3, Hsd3b7, Ifitm1, Gas6, Plat, Man2b1, B3gnt9, S1pr2, Anxa2, Shisa5, Tcf21, Smpdl3a, Sptbn1, Stc2, Scpep1, Prcd, Npc2, Dbn1, Arsb, Adk, Pdlim2, Fndc3a, C7, Acvrl1, Cfb, Cdc42ep2, Scd1*	*Ackr3, Col6a3, ENSMUSG00000090208, Prelp, Klf4, Bhlhe40, Pdgfrl, Nfix, Ifngr1, Ace, Cpq, Enpp2, Adamts5, Srpx, Srpx2*	*Hmgcs2, Sfrp1, Igfbp4, Qpct*	*Cyp27a1, Vcam1, Apoe, Rbp1, Lama4, Ifi27l2a, Txndc5*	*Hmcn2, Thbs3, Ssc5d, Itga11, Cilp, Scara3*	*Tram1, Col3a1, Col5a2, Fmo1, Plxdc2, Cd302, Rcn1, Cpxm1, Lbp, Ctsk, B4galt1, Tgfbr3, Pcolce, Kdelr2, Inmt, Tmsb10, Oaf, Hexa, Tmed3, Akap12, Lama2, Col6a2, Col6a1, Rab34, Lgals9, Mrc2, Cygb, Lgals3bp, Osr1, Nid1, Ogn, Ctsl, Hexb, Mmp14, Selenop, Lima1, Apod, Boc, Tmem204, ENSMUSG00000092511, Lrrn4cl, Rab3il1*	*Pid1, Cd55, Fmo2, Itih5, Cercam, Ptgis, ENSMUSG00000097249, Fzd1, Sema3c, Slco2b1, Plekho2, Efemp1, Gas7, Serpina3n, Vcan, C1qtnf3, Il33*	*Penk, Pcsk6, Islr*	*Sulf1, Steap3, Podn, Mfap2, Vwa1, Fxyd6, Igf1, Gpx3, Pmp22, Timp2, Lamb1, Tgfbi, Gas1, Bmp1, Itgbl1, Ly6e, Abi3bp, Smoc2, Ms4a4d, Itm2a*	*Sulf2, Mmp23, Spon2, Matn2, Col8a1*	*Fn1, Pamr1, Fbn1, F3, Adh1, Svep1, Pdpn, Adgrd1, Emp1, Mgp, Ltbp4, Nupr1, Tgfbr2, Clec3b, Man1a, Lrp1, Aebp1, Fbln5, Ahnak2, Bmp4, Col14a1, Ly6c1, Fndc1, Thbs2, Tnxb, Xdh, Colec12, Pcdhgc3, Cd248, Add3, Ablim1*	*Nav1, Dpt, Hsd11b1, Cd34, Celf2, Entpd2, Col5a1, Gsn, Slc43a3, S100a16, S100a10, Olfml3, Lpar1, Htra3, Ugdh, Pdgfra, ENSMUSG00000107314, Medag, Col1a2, Fbln2, Mfap5, Mgst1, Lsp1, Mmp2, Dpep1, Loxl1, Pcolce2, Bicc1, Dcn, Lum, Gfpt2, Adamts2, Mfap4, Serpinf1, Col1a1, Abca8a, Rnase4, Scara5, Ly6a, Fbln1, Igfbp6, Heg1, Ccdc80, Pi16, C3*
Mural cell genes	*Tns1, Foxs1, Fabp4, Serpini1, Steap4, Art3, Cav1, Ccnd2, Arhgdib, Kcnj8, Abcc9, Imp3, Sept4, Higd1b, Colec11, Vcl, Lpp, Fam162a, Cd200, Pdgfrb*	*Sbspon, Lrrfip1, Sdhc, Ndufs2, Atp1a2, Crat, Pde1a, Tgm2, ENSMUSG00000092283, Gyg, Plekho1, Tspan2, Arhgap29, Bcar3, Ccdc107, Tmem38b, Tmem51, Wdr1, Aqp1, Mgll, Kcna5, Ldhb, Ppp1r12c, Gpi1, ENSMUSG00000097660, Synm, Idh2, Unc45a, Lrrc32, Olfr78, St5, Tgfb1i1, Inpp5a, Npy1r, Jam3, Usp2, Filip1, Klhdc8b, Perp, Chchd10, Cnn2, Csrp2, Syngr2, Hist1h1c, Pdlim7, Ldb3, Cyc1, Fez2, Mamdc2*	*Myo1b, Klhl23, ENSMUSG00000106890, Btbd3, Inpp4b, Bcr, Gjc1, Syne2, Gcnt2, Itga1, P3h2, Tmem50b, Cdc42ep3, Rock1*	*Kcne4, Ramp1, Btg2, 4930523C07Rik, Atf3, ENSMUSG00000097770, Olfml2a, Id1, Cebpb, Rap1a, Efhd2, Cdk11b, Fosl2, Coro1c, Pdgfa, ENSMUSG00000085382, Lmcd1, Zfp36, Ppp1r15a, Cpxm2, Slc7a2, Rrad, Cryab, Csrnp1, Utrn, Gadd45b, Nudt4, Rasd1, Epn2, Blmh, Prkar1a, Socs3, Id2, Dgkb, Net1, Tsc22d1, Amigo2, Nr4a1, Hes1, Phldb2, Adamts1, Ets2, Ltbp1, Pls3*	*ENSMUSG00000090996, Gucy1a1, Casq2, Atp2a2, Mfge8, Oaz2, Kcnmb1, Kank1, Slc25a5*	*Rgs5, Gng11, Ano1, Mtus1, Snrk, Vtn*	*Ccdc3, Cald1, Lims1, Clu*	*Heyl, Nr2f2, Lgalsl, Asb2, Pygm, Tspan7, Dmd*	*Arid5a, Speg, Csrp1, Itga8, Cd82, Dstn, Trp53inp2, Lama5, Kcnab1, Synpo2, Hspb7, Tesc, Hspb1, Atp6v0e2, Grip2, Stk38l, Actn4, Ppp1r14a, Hspb6, Pnpla2, Palld, Wfdc1, Fbxl22, Susd5, Myl6, P2rx1, Abr, Sgca, Gprc5c, Itpk1, Ckb, Rgs7bp, Camk2g, Prkcd, Pck2, Myom1, Mapre2, Fgf1, Marveld1*	*Rcsd1, Gm13889, Tm4sf1, Errfi1, Cav2, Actg2, Angpt2, ENSMUSG00000098650, B3gnt2, 2010111I01Rik, Map1b*	*Gucy1b1, Olfr558, Cog7, Ndufa4l2*	*Dbndd2, Pbxip1, Pde3a, Rbpms, Ednra, Smtn, ENSMUSG00000087291, Mustn1, Entpd1*	*Rasl11a*	*Map3k20, Trp53i11, Parm1, ENSMUSG00000108011, Nrip2, Dmpk, Tcim, Pdlim3, Cpe, Nkd1, Pln, Myocd, Slc2a4, Atp2a3, Cap2, Slmap, Ndrg2, Gpr20, Tuba1c, Lbh, Sorbs1*	*Des, Ppp1r12b, Lmod1, Mgst3, Rgs4, Pcp4l1, Stom, Cox4i2, Myl9, Pkig, Tpm2, Gja4, Tinagl1, Ppp1cb, Bcam, Uba2, Arhgef17, Mrvi1, Mob2, Sorbs2, Cnn1, Esam, Mcam, Tagln, Cspg4, Rasl12, Rbpms2, Tpm1, Ppp1r12a, Itga7, Atp1b2, S1pr3, Mef2c, Sncg, Ptp4a3, Myh11, Mylk, Filip1l, Notch3, Rcan2, Epas1, Cystm1, Rasgrp2, Acta2, Flna*

Inter-organ cross reference of genes specifically over-represented in firboblasts or mural cells, per organ. The respective overlap of enriched genes for the different organ combinations are shown. Compare to venn diagrams displayed in Figure 1f.

Supplementary Table 2: **Skeletal muscle and heart specific FB cross-reference.**

differential expression
log2 FC >2

heart valve interstitial cells	skeletal muscle perimysial cells	intersection
Stk17b, Prelp, Shisa4, Atp1b1, Frzb, Pamr1, Cd44, Paqr6, Npr1, Lmo4, Sdc3, Ptn, Dkk3, Cyp2e1, Cadm1, Tcf12, Pxylp1, Meox1, Ltbp2, Scara3, Clu, Smim3, Papss2, Tspan7	*Rgs2, ENSMUSG00000097770, Ccdc3, Arl5a, Fibin, Fbln7, Ptgis, Col11a1, Tnc, Ptprd, Col8a2, Fzd1, Pdgfa, Slc6a6, Clec11a, Gas2, Scube2, ENSMUSG00000086814, Cilp2, Fxyd6, Col12a1, Sema3b, Calhm5, Kera, Tns3, Mfap4, Rflnb, Myo1d, Chad, Thbs4, Lhfpl2, Emb, Itgbl1, P3h2, Chodl, Thbs2, Ptx4, Col11a2, Mkx, Lox, Zfp185, Tnmd*	*1500015O10Rik, Fmod, Prg4, Thbs1, Angptl7, Lmcd1, Cpxm2, Comp, Wif1, Abi3bp*

Heart and skeletal muscle cross-reference of genes specifically over-represented in the fibroblast subtypes of perimysial cells or cardiac valve interstitial cells, respectively. Compare to venn diagram displayed in Figure 4e.

Supplementary Table 3: **Colon and bladder specific FB cross-reference.**

differential expression
log2 FC >2

**colon Tnc+ Cd34-**	**bladder Tnc+ Cd34-**	**intersection**
Prex2, Trpa1, Il1r1, Il1rl1, Rgs2, Pla2g4a, Ptgs2, Mpzl1, ENSMUSG00000097770, Ccdc3, Syt13, Cd82, Galk2, Fgf7, Smox, Bmp2, Id1, Procr, Fam210b, Lama5, Slco4a1, Fat4, P2ry1, Lxn, Iqschfp, Schip1, Lef1, Lpar3, Tox, Gem, Plin2, Rab3b, Slc2a1, Ece1, Wfs1, Cd38, Pdgfra, Ereg, Dmp1, Lrrc8c, Tbx3, Akr1b8, Bpgm, Eva1a, Slc6a6, Ano2, Ntf3, C5ar1, Fosb, Ctsc, Ucp2, Insc, Rgs10, Nrg1, ENSMUSG00000097928, Dnajb1, Adgre5, Sall1, Nqo1, Cdh13, Foxl1, Agt, Col6a4, Sema3f, Ptprk, Gja1, Lgr5, Ptprr, B4galnt1, Emid1, Glp2r, Ttyh2, Cd300e, Ahr, Bdkrb2, Nedd9, Tgfbi, Serinc5, F2r, Plau, Wnt5a, Fgf9, Ednrb, Abcc4, Otulinl, Wisp1, Dll1, Ier3, Pla2g7, Arhgap28, Kazald1, Timp1, Nxf7, Col4a5	*Adam23, Fn1, Col6a3, F11r, Calcrl, Tgm2, ENSMUSG00000092283, Gata5, Nkain4, Car3, Sfrp2, Dkk2, Col16a1, Cpz, Bmp3, Hoxa11, Spon1, Il4ra, Palld, Hhip, Nkd1, Neo1, Thsd4, Itga11, Aldh1a2, Col12a1, Prss35, Cck, Tspan8, Avpr1a, Myh10, Notum, Ptch1, Vcan, Armh4, Slc7a8, Myh9, Mc5r, Slc14a1, Myof, Rbp4, Srpx*	*Ptgs1, Cd44, Bmp7, Tnc, Rerg, Abhd2, Bmp5, ENSMUSG00000097836, Tspan13, Cxcl14, Plk2, Twsg1, Srpx2*

Colon and bladder cross-reference of genes specifically over-represented in the fibroblast subtypes of sub-epithelial located fibroblasts. Compare to venn diagram displayed in Figure 6e.

Supplementary Table 4: **Cells from individual mice per pagoda2 cluster (complete dataset).**

	individual mice																								
**cluster**	***1***	***2***	***3***	***4***	***5***	***pool of two mice, same as 5 & 8***	***6***	***7***	***8***	***9***	***10***	***11***	***12***	***13***	***14***	***15***	***16***	***17***	***18***	***19***	***20***	***21***	***22***	***23***	***24***
**1**	1	0	212	2	1	2	4	0	0	0	3	0	5	3	21	2	3	1	90	3	0	273	0	0	245
**2**	0	0	1	1	18	22	1	0	16	0	0	0	0	1	2	57	16	0	8	0	0	2	41	0	0
**3**	1	1	0	1	5	7	1	0	5	0	1	0	0	57	0	8	0	0	1	1	0	0	0	0	0
**4**	0	0	0	0	58	91	0	0	60	0	1	0	0	2	1	110	77	2	43	0	1	0	68	0	0
**5**	0	0	1	31	0	0	1	24	0	1	112	47	0	0	8	2	1	87	93	96	0	1	0	0	0
**6**	0	2	122	97	1	1	1	1	2	0	4	0	2	0	59	1	0	2	21	4	0	34	4	1	0
**7**	90	8	0	1	0	0	175	1	0	0	289	4	141	99	1	143	0	114	1	3	0	0	0	0	0
**8**	53	1	0	0	0	0	56	0	0	0	66	0	39	12	0	18	0	5	0	0	0	0	0	0	0
**9**	2	0	0	1	14	36	1	0	28	0	1	1	0	40	1	119	102	0	16	1	0	0	99	0	0
**10**	0	0	0	1	20	17	0	0	0	0	0	0	0	20	1	53	38	0	37	0	2	1	150	0	0
**11**	0	0	0	2	0	0	0	0	0	0	0	0	0	0	0	0	0	40	12	2	0	0	0	0	0
**12**	0	0	1	36	0	1	3	9	0	10	102	59	2	1	14	3	3	69	175	128	0	0	3	0	2
**13**	0	0	0	8	0	0	0	37	0	0	14	44	0	0	2	0	0	45	9	19	0	0	0	0	0
**14**	0	0	0	0	0	0	0	0	0	0	0	0	0	0	33	1	0	1	4	0	0	2	0	0	0
**15**	0	0	21	0	0	0	0	0	0	0	0	0	0	0	3	0	0	0	27	0	0	16	0	0	4
**16**	0	14	6	71	1	4	19	0	2	0	12	0	1	123	108	33	23	22	99	1	4	1	1	0	0

Summary of cell number captured from individual mice and their distribution in the pagoda2 clusters of the complete dataset, compare to Supplementary Figure 1c.

Supplementary Table 5: **Antibodies and RNAscope probes.**

Antibodies:

Name (protein / antigen)	gene	Company	Reference	host	clone	Lot	Dilution used	Comment	Vaidation
alpha-SMA	*Acta2*	Sigma	C6198	mouse	1A4	037M4783V	1:500	Cy3-conjugated	more than 500 reference publications
CD31	*Pecam1*	R&D Systems	AF3628	goat		YZU0114021	1:300		more than 80 reference publications
CD31	*Pecam1*	BD Bioscience	550274	rat	MEC13.3	53198	1:100		7 references / recommended for IHC
CD31	*Pecam1*	BD Bioscience	561814	rat	MEC13.3	7096610	1:200	APC-conjugated for FACS	13 references / recommended for Flow
CD31	*Pecam1*	BD Bioscience	561813	rat	MEC13.3	7159852	1:100	FITC-conjugated for FACS	same as above
CD34	*Cd34*	Invitrogen	MA1-22646	rat	MEC14.7	TD2554478E	1:100		2 references (Flow) / recommended for IHC
CNN1	*Cnn1*	Abcam	ab216651	rabbit	EP798Y	GR3196369-3	1:300		38 references / recommended for IHC
COX-2	*Ptgs2*	Cell Signalling	12282	rabbit	D5H5	4	1:200		more than 100 references / recommended for IHC
Cytochrome P450 2E1	*CYP2E1*	Novus	NBP1-85367	rabbit		B115936	1:100		one reference / recommended for IHC
HHIP	*Hhip*	R&D Systems	AF1568	goat		JEK021911B	1:100		4 references (2 for IHC)
NG2	*Cspg4*	Millipore	AB5320	rabbit		LV1519145	1:300		more than 600 references / recommended for IHC
NGFR	*Ngfr*	abcam	ab52987	rabbit	EP1030Y	GR3238403-3	1:100		recommended for IHC
PDGFRalpha	*Pdgfra*	R&D Systems	AF1062	goat		HMQ0216021	1:200		more than 50 references / recommended for IHC
PDGFRalpha	*Pdgfra*	eBioscience	17-1402-82	rat	APA5	4303763	1:200	APC-conjugated for FACS	15 references / recommended for Flow
PDGFRbeta	*Pdgfrb*	R&D Systems	AF1042	goat		GOV04161 / GOV0417041	1:200		27 references / recommended for IHC
PDGFRbeta	*Pdgfrb*	ebioscience	17-1401-81	rat	APB5	4303343	1:200	APC-conjugated for FACS	more than 60 references / recommended for Flow
POSTN	*Postn*	R&D Systems	MAB3548	rat	345613	YCF041807B	1:200		4 references / recommended for IHC
ST2 / IL33R	*Il1rl1*	R&D Systems	AF1004	goat		KDZ021804A	1:100		6 references (one for IHC)
TNC	*Tnc*	R&D Systems	MAB2138	rat	578	KLC071802	1:200		8 references / recommended for IHC
TSP4	*Thbs4*	R&D Systems	MAB7860	rat	893655	CIPM011807A	1:200		recommended for IHC
Type VIII collagen alpha-1	*Col8a1*	Novus	NBP2-13856	rabbit		C107088	1:100		recommended for IHC
Type XXII collagen alpha-1	*Col22a1*	Novus	NBP1-91056	rabbit		A104177	1:100		one reference / recommended for IHC
VWF	*Vwf*	DAKO	A0082	rabbit		20062242	1:300		more than 600 references / recommended for IHC
WIF1	*Wif1*	R&D Systems	AF135	goat		EUQ021901A	1:100		one reference
goat IgG	*	Invitrogen	A21082	donkey		1889311	1:500	AlexaFluor-633 conjugated	
goat IgG	*	Invitrogen	A21432	donkey		1818686	1:500	AlexaFluor-555 conjugated	
rabbit IgG	*	Invitrogen	A10043	donkey		1917929	1:500	AlexaFluor-680 conjugated	
rabbit IgG	*	Invitrogen	A31572	donkey		1837922	1:500	AlexaFluor-555 conjugated	
rat IgG	*	Invitrogen	A21208	donkey		1900239	1:500	AlexaFluor-488 conjugated	
rat IgG	*	Invitrogen	SA5-10029	donkey		RK2304679	1:500	AlexaFluor-647 conjugated	
rat IgG	*	Jackson Laboratories	712-165-153	donkey		139289	1:300	Cy3-conjugated	

*secondary antibody

RNAScope:

Probe (mouse gene)	type	Lot	Reference
Mm-Pdgfra	C2	18017C	480661-C2
Mm-Col12a1	C1	18017A	312631
Mm-Dcn	C3	18017C	413281-C3
Mm-Col1a1	C1	18017A	319371
Mm-Tnc	C2	18071B	465021-C2
Mm-Foxl1	C1	19010A	407401
Mm-Pecam1	C3	18071C	316721-C3
Mm-Wif1	C1	18079A	412361
**Reagents**	**type**	**Lot**	**Reference**
Protease IV	pre-treat	2002756	322336
detection Reagents	fluorescent	2002259	320851
