# Supplementary material for: Single-cell analysis uncovers fibroblast heterogeneity and criteria for fibroblast and mural cell identification and discrimination
Source: Nat Commun. 2020 Aug 7;11:3953. doi: 10.1038/s41467-020-17740-1 (PMC7414220; doi:10.1038/s41467-020-17740-1)
Supplement: Supplementary file 3 — Description of Additional Supplementary Files [file 41467_2020_17740_MOESM3_ESM.docx]

To be included in the cover letter.

Supplementary Data legends

Supplementary Movie 1: 3-dimensional-rendering of immunofluorescent staining of a sub-epithelial capillary loop close to the colonic surface with pericytes attached at the far side of the capillary. Compare to Supplementary Movie 2. Gray = DAPI; magenta = CD31; cyan = *Pdgfrb^GFP^*, frame size = 116 x 116 µm.

Supplementary Movie 2: Slice-by-slice walkthrough to the same image shown as 3-deminsional rendering in Supplementary Movie 1. Gray = DAPI; magenta = CD31; cyan = *Pdgfrb^GFP^*, frame size = 116 x 116 µm.

Supplementary Data 1: Cell number per pagoda2 cluster, and cellular origin by organ of cells in the respective pagoda2 cluster shown for the complete dataset (upper panel), the mural cell dataset (middle panel) and the combined dataset (lower panel).

Supplementary Data 2: Lists of genes compiled for gene-set analysis and UMAP visualization. All genes within the respective GO-term or gene-list are shown, as well as the genes expressed in the complete dataset, which were used for UMAP visualization. Compare to Figure 2c.
