# Supplementary material for: Single-cell analysis uncovers fibroblast heterogeneity and criteria for fibroblast and mural cell identification and discrimination
Source: Nat Commun. 2020 Aug 7;11:3953. doi: 10.1038/s41467-020-17740-1 (PMC7414220; doi:10.1038/s41467-020-17740-1)
Supplement: Supplementary file 8 — Reporting Summary [file 41467_2020_17740_MOESM8_ESM.pdf]

nature research

Corresponding author(s): Lars Muhl, Christer Betsholtz

Last updated by author(s): Jun 16, 2020

Reporting Summary

Nature Research wishes to improve the reproducibility of the work that we publish. This form provides structure for consistency and transparency in reporting. For further information on Nature Research policies, seeAuthors & Referees and theEditorial Policy Checklist .

Statistics

For all statistical analyses, confirm that the following items are present in the figure legend, table legend, main text, or Methods section.

n/a	Confirmed	
☐	☒	The exact sample size (*n*) for each experimental group/condition, given as a discrete number and unit of measurement
☐	☒	A statement on whether measurements were taken from distinct samples or whether the same sample was measured repeatedly
☐	☒	The statistical test(s) used AND whether they are one- or two-sided<br>*Only common tests should be described solely by name; describe more complex techniques in the Methods section.*
☒	☐	A description of all covariates tested
☒	☐	A description of any assumptions or corrections, such as tests of normality and adjustment for multiple comparisons
☒	☐	A full description of the statistical parameters including central tendency (e.g. means) or other basic estimates (e.g. regression coefficient) AND variation (e.g. standard deviation) or associated estimates of uncertainty (e.g. confidence intervals)
☐	☒	For null hypothesis testing, the test statistic (e.g. *F*, *t*, *r*) with confidence intervals, effect sizes, degrees of freedom and *P* value noted<br>*Give P values as exact values whenever suitable.*
☒	☐	For Bayesian analysis, information on the choice of priors and Markov chain Monte Carlo settings
☒	☐	For hierarchical and complex designs, identification of the appropriate level for tests and full reporting of outcomes
☒	☐	Estimates of effect sizes (e.g. Cohen's *d*, Pearson's *r*), indicating how they were calculated

Our web collection on statistics for biologists contains articles on many of the points above.

Software and code

Policy information about availability of computer code

Data collection

no software was used for data collection

Data analysis

For RNA-sequence processing the following software was applied: Illumina pipeline (bcl2fastq 2.19.0.316), TopHat (version 2.1.1) with Bowtie1 (version 1.1.2), or Bowtie2 (version 2.2.6) option, trim galore (version 0.4.4), samtools (version 1.9), and featureCounts (version 1.4.6-p5).
For data analysis in this study the following software was applied: BackSPIN https://github.com/linnarsson-lab/BackSPIN) for cell clustering, R-software package SingleCellExperiment (version 1.4.1), R-software package monocle (version 2.10.1). For data visualization in 2-dimensional UMAP plots and for heat maps the incorporated functions in the SingleCellExperiment R-package were applied. Additionally, for heat maps the R-package pheatmap (version 1.0.12) was used . Venn diagrams were prepared using the R-software package VennDiagram (version 1.6.20).
For immunofluorescence and RNAscope micrograph capture Leica Application Suite X version 3.5.6.21594 was used, for image processing and handling FIJI/ImageJ version 2.0.0-rc-68/1.52e and Adobe Illustrator CC 2018 were used.

For manuscripts utilizing custom algorithms or software that are central to the research but not yet described in published literature, software must be made available to editors/reviewers. We strongly encourage code deposition in a community repository (e.g. GitHub). See the Nature Research guidelines for submitting code & software for further information.

Data

Policy information about availability of data

All manuscripts must include a data availability statement. This statement should provide the following information, where applicable:
- Accession codes, unique identifiers, or web links for publicly available datasets
- A list of figures that have associated raw data
- A description of any restrictions on data availability

The data analyzed in this study are made available as a searchable database of gene - by - gene bar plots accessible at https://betsholtzlab.org/Publications/

FibroblastMural/database.html. The raw data is deposited in the NCBI Gene Expression Omnibus database with the accession number GSE150294.

Field-specific reporting

Please select the one below that is the best fit for your research. If you are not sure, read the appropriate sections before making your selection.

☒ Life sciences ☐ Behavioural & social sciences ☐ Ecological, evolutionary & environmental sciences

For a reference copy of the document with all sections, see nature.com/documents/nr-reporting-summary-flat.pdf

Life sciences study design

All studies must disclose on these points even when the disclosure is negative.

Sample size	In this study, we aimed to sequence as many perivascular mesenchymal single cells as possible and achieved in total 6158 cells of high quality from the heart (1279 cells), skeletal muscle (1754 cells), colon (1646 cells) and bladder (1479 cells). These cells were collected from a total of 24 different male mice. No statistical methods were used to predetermine sample size. Sample size was not predetermined, but adjusted according to the identity of the captured cells. Sufficient sample size was determined by previous experience and in accordance to previouslty published literature.
Data exclusions	We have used the following predetermined cell exclusion criteria that are based on previous experience to yield a dataset of high quality single cell transcriptomes: cells with total aligned reads less than 50,000 were removed, also cells with a percentage of reads aligned to ERCC (technical spike-in control) or mitochondrial genes higher than 10% were removed, and cells with less than 1500 detected genes were removed.<br>In order to focus on the mesenchymal cell population, cells that do not express either Pdgfra or Pdgfrb, but were unintentionally collected were also removed from the dataset. Those cells usually expressed typical marker genes of either endothelial cells (Pecam1, Cdh5), immune cells (Ptprc, Cd68) or epithelial cells (Cdh1, Epcam).
Replication	The gene expression profiles reported herein are observed from dozens to hundreds of individual cells that were collected from 24 individual animals and at different time points. All experimental findings were reliably reproduced.
Randomization	Mice were selected for cell isolation based on sex (male), age ((10 - 20 weeks old, adult) and genotype (for cell sorting of fluorescent reporter expression). For only antibody based cell sorting random C57Bl6/J male mice at corresponding age from the core breeding were selected. All other criteria were not considered and as such, randomized.
Blinding	The selection of appropriate mice (sex, age, genotype) was completely random, while in this study only male mice were selected. The selection of cell-capture was dependent on fluorescent reporter expression or antibody staining, however, the exact identity of the selected cells was unknown to the researched until after the transcriptome analysis. The clustering of the single cell data was performed using the pagoda2 algorithm, which is unbiased. No predetermination of groups was done, therefore, no blinding was necessary.

Reporting for specific materials, systems and methods

We require information from authors about some types of materials, experimental systems and methods used in many studies. Here, indicate whether each material, system or method listed is relevant to your study. If you are not sure if a list item applies to your research, read the appropriate section before selecting a response.

Materials & experimental systems

n/a	Involved in the study	
☐	☒	Antibodies
☒	☐	Eukaryotic cell lines
☒	☐	Palaeontology
☐	☒	Animals and other organisms
☒	☐	Human research participants
☒	☐	Clinical data

Methods

n/a	Involved in the study	
☒	☐	ChIP-seq
☐	☒	Flow cytometry
☒	☐	MRI-based neuroimaging

Antibodies

Antibodies used	All antibodies that were used in the study are provided in supplementary table 5
Validation	The usage of antibodies for immunofluorescence in this study was solely for confirmation of transcriptomic features that were observed in the single-cell RNA-seq analysis. Antibody staining was performed in conjunction with well established fluorescent reporter mouse models (see below: Laboratory animals). Therefore, the study does not claim conclusion about cell identity only derived from antibody staining properties.<br>The specifics about each antibody used in the study are summarized in supplementary table 6. Further information about antibody specificity, species cross-reactivity, recommended applications and links to relevant publications can be found on the

manufacturers' website.
Information about the used antibodies:
- anti alpha-SMA, Sigma (C6198) is recommended for IHC and has more than 500 references
- anti CD31, R&D Systems (AF3628) is recommended for IHC and has more than 80 references
- anti CD31, BD Bioscience (550274) is recommended for IHC and has seven references
- anti CD31-APC /-FITC, BD Biosciences (561814 / 561813) are recommended for Flow and have 13 references
- anti CD34, Invitrogen (MA1-22646) has two references (Flow) and is recommended for IHC
- anti CNN1, Abcam (ab216651) has 38 references and is recommended for IHC
- anti COX-2, Cell Signalling (12282) has more then 100 references and is recommended for IHC
- anti Cytochrome P450 2E1, Novus (NBP1-85367) has one reference and is recommended for IHC
- anti HHIP, R&D Systems (AF1568) has four references (2 for IHC)
- anti NG2, Millipore (AB5320) has more than 600 references and is recommended for IHC
- anti NGFR, Abcam (ab52987) is recommended for IHC
- anti PDGFRalpha, R&D Systems (AF1062) has more than 50 references and is recommended for OHC
- anti PDGFRalpha-APC, eBioscience (17-1402-82) has 15 references and is recommended for Flow
- anti PDGFRbeta, R&D Systems (AF1042) has 27 references and is recommended for IHC
- anti PDGFRbeta-APC, eBioscience (17-1401-81) has more than 60 references and is recommended for Flow
- anti POSTN, R&D Systems (MAB3548) has four references and is recommended for IHC
- anti ST2/IL33R, R&D Systems (AF1004) has six references (one for IHC)
- anti TNC, R&D Systems (MAB2138) has eight references and is recommended for IHC
- anti TSP4, R&D Systems (MAB7860) is recommended for IHC
- anti Type VIII collagen-alpha-1, Novus (NBP2-13856) is recommended for IHC
- anti Type XXII collagen-alpha-1, Novus (NBP1-91056) is recommended for IHC
- anti VWF, Dako (A0082) has more than 600 references and is recommended for IHC
- anti WIF1, R&D Systems (AF135) has no reference and is recommended for western blotting

Animals and other organisms

Policy information about studies involving animals; ARRIVE guidelines recommended for reporting animal research

Laboratory animals	In this study we used male mice at the age of 10 - 20 weeks from the following strains; C57Bl6 (The Jackson Laboratory, C57Bl6/J, maintained as breeding colony at the local animal facility), PdgfrbGFP (Genesat.org, Tg(Pdgfrb-eGFP) JN169Gsat/Mmucd), PdgfraH2BGFP ((Pdgfratm11(EGFP)Sor), a gift from P. Soriano), Cspg4dsRED (The Jackson Laboratory, Tg(Cspg4-DsRed.T1)1Akik/J), Acta2GFP (The Jackson Laboratory, Tg(Acta2-GFP)1Pfk), Cldn5GFP (Tg(Cldn5-GFP)Cbet/U), and combinations of these strains. The mice were housed in single ventilated cages with a 12 h light - 12 h dark cycle with access to chow and water ad libitum. The temperature was kept at 20 ±2°C and the relative humidity was kept at 50 ±5%.
Wild animals	The study does not include wild animals.
Field-collected samples	The study does not contain field-collected samples.
Ethics oversight	The mouse experiments for this study were carried out in accordance to the local guidelines and regulations for animal welfare (Linköpings Animal Research Ethics committee [Linköpings djurförsöksetiska nämnd], approved Ethical number ID 729).

Note that full information on the approval of the study protocol must also be provided in the manuscript.

Flow Cytometry

Plots

Confirm that:

- [x] The axis labels state the marker and fluorochrome used (e.g. CD4-FITC).
- [x] The axis scales are clearly visible. Include numbers along axes only for bottom left plot of group (a 'group' is an analysis of identical markers).
- [x] All plots are contour plots with outliers or pseudocolor plots.
- [x] A numerical value for number of cells or percentage (with statistics) is provided.

Methodology

Sample preparation	A detailed description of the cell isolation and sample preparation can be found in the material and methods section of the supplementary information file.
Instrument	BD FACSAria III or FACSMelody
Software	FACS Diva v8.0.2 (FACSAria III), FACS Chorus 1.3, Application data vers. 1.1.18.0 (FACSMelody), FlowJo v10

Cell population abundance

Cell fraction purity is assessed from the single cell transcriptomes as described in the manuscript. The cell sorting by FACS was not used for cell type identification, but for enrichment and capture of cell populations for single cell RNA-sequencing. Therefore, the percentage abundance of distinct cell populations identified by flow cytometry has no value for cell identity definition or interpretation. Cell population numbers can vary between each cell isolation and between the preparations from the different organs. However, this was irrelevant since the sorting was used to enrich for the target cell populations, marked with antibodies (PDGFRalpha or PDGFRbeta) or by fluorescent reporter constructs.

Gating strategy

The gating strategy is explained in detail in the material and methods section. For each of the different reporter mouse strains the same gating strategy as exemplified in Supplementary Figure 1a was applied and adjusted to the specific requirements of the reporters. The additional staining with anti-CD31 antibody was used to exclude endothelial cell contaminated (whole cell or cell fragments) mural cells or fibroblasts to be selected and sorted into the 384-well plate.

☒ Tick this box to confirm that a figure exemplifying the gating strategy is provided in the Supplementary Information.
